# Supplementary material for: Deletion of wfs1 Impairs Oligodendrocyte Precursor Cells Dorsal Distribution and Myelination Through the wfs1-hmgcs1 Axis in Zebrafish
Source: Biology (Basel). 2026 Aug 16;15(16):1402. doi: 10.3390/biology15161402 (PMC13510151; doi:10.3390/biology15161402)
Supplement: Supplementary file 1 [file biology-15-01402-s001.zip › File S1-Transcriptome sequencing data and differentially expressed genes (DEGs) in wfs1-- zebrafish at 5 dpf/2.KEGG/wfs1KOvsWT/DOWN/src/dre01240.html]

dre01240


- dre:100004977

- Down regulated genes

100004977\_2(-3.12630634600606)

- dre:100004977

- Down regulated genes

100004977\_2(-3.12630634600606)

- dre:64607

- Down regulated genes

64607\_1(-2.39285410667541)

- dre:334195

- Down regulated genes

334195(-1.02556051088404)
- dre:678634

- Down regulated genes

678634(-2.56566805889366)

- dre:100007686

- Down regulated genes

100007686(-1.06018652619591)

- dre:406731

- Down regulated genes

406731(-3.65881284610119)
- dre:678597

- Down regulated genes

100415796(-3.10645904576552)
- dre:570913

- Down regulated genes

570913\_1(-2.18577817117659)
- dre:767756

- Down regulated genes

767756(-1.81780175682628)

- dre:445326

- Down regulated genes

445326(-1.62876055255802)

- dre:403070

- Down regulated genes

403070(-1.45615173353575)

- dre:100536795

- Down regulated genes

100536795(-1.64304185838866)

- dre:407663

- Down regulated genes

407663(-1.06564682717154)

Close
